# Consciousness, 4E cognition and Aristotle: a few conceptual and historical aspects

**DOI:** 10.3389/fncom.2023.1204602

**Published:** 2023-11-21

**Authors:** Diana Stanciu

**Affiliations:** Romanian Young Academy (hosted by the University of Bucharest), Bucharest, Romania

**Keywords:** consciousness, the mind-body problem, neurophysiological phenomena related to consciousness, brain networks dynamics, 4E cognition, Aristotle, active intellect, causality

## Abstract

The new approach in cognitive science largely known as “4E cognition” (embodied/embedded/enactive/extended cognition), which sheds new light on the complex dynamics of human consciousness, seems to revive some of Aristotle's views. For instance, the concept of “nature” (*phusis*) and the discussion on “active intellect” (*nous poiêtikos*) may be particularly relevant in this respect. Out of the various definitions of “nature” in Aristotle's *Physics, On the Parts of Animals* and *Second Analytics*, I will concentrate on nature defined as an inner impulse to movement, neither entirely “corporeal,” nor entirely “incorporeal,” and neither entirely “substantial,” nor entirely “accidental.” Related to that, I will consider the distinction in *On the Soul* between the “active” and the “passive” intellect, which Aristotle asserted as generally present in “nature” itself. By offering a conceptual and historical analysis of these views, I intend to show how the mind–body problem, which is essential for the explanation of consciousness, could be somewhat either eluded or transcended by both ancients and contemporaries on the basis of a subtle account of causation. While not attempting to diminish the impact of the Cartesian paradigm, which led to the so-called “hard problem of consciousness,” I suggest that the most recent neuroscience discoveries on the neurophysiological phenomena related to human consciousness could be better explained and understood if interpreted within a 4E cognition paradigm, inspired by some Aristotelian views.

## Introduction

This is a conceptual and historical analysis suggesting that new scientific studies on the neurophysiological phenomena and the neural network dynamics related to consciousness can be better explained and understood within an interdisciplinary context inspired by the new 4E cognition (embodied/embedded/enactive/extended) views. These scientific and cognitive/philosophical approaches are deeply related and can support each other in the study of consciousness. But, in fact, the 4E cognition does not offer a completely new view on human cognition. Instead, it actually synthesizes various ancient, modern, and contemporary ideas that were in tune with the developments of science in those times and are very much in tune with the new developments in neuroscience nowadays as well.

For instance, the early modern empiricist philosophers brought much innovation in the theories of human cognition and that was related to the new development of experimental sciences at that time. This development inspired the empiricists to challenge the rationalist Cartesian paradigm and emphasize the importance of experience in cognition. They actually considered all knowledge to derive from sensorial data, a view that had several antecedents in antiquity—especially among the Empiricists, Stoics, and Pyrrhonists and also among the Greek medical schools.

While acknowledging all this development, but not concentrating on it, this article will only try to emphasize the inspiration contemporary 4E cognition proponents, as well as the early modern empiricists, found in ancient theories of cognition. Moreover, here I will concentrate on Aristotle, who provided a synthetic and still innovative conception of the human “soul”—especially regarding its empirical substratum, emotions, consciousness, and agency. Aristotle's views on *phusis* (nature), *phantasiai* (phenomena/experience of the way things appear to us/information received from sense experience), and *phantasmata* (perception or the act of sensing) as important and related to the thinking processes developed by the active intellect (*nous poiêtikos*) in cognition will receive a somewhat detailed analysis that may also be revealing for the 4E cognition views on human consciousness and its relationship to the world around^1^.

The article will also try to demonstrate that some of Aristotle's ancient views are still relevant for scientific research nowadays—especially for those specialists trying to establish the principles and modalities of our thought, consciousness, and agency functioning in relation to better and better researched and understood neurophysiological phenomena underlying them. I am trying to generally suggest that it is only through an interdisciplinary approach, in which various views and developments cross-fertilize, that we can more comprehensively understand the mind–body problem in the years to come.

Very important in this respect are, in my view, Aristotle's explanations of causation. They offer a subtle view in which causes are deemed to be not only material and formal but also efficient and final. Moreover, even if this does not solve the Cartesian mind–body problem, it does place it in a wider and more comprehensive context. Beyond the question of whether the body causes the mind as its material cause or the mind causes the body as its formal cause and beyond the idea of efficient causation supposed to bridge the mind–body gap and explain the interaction of two ontologically different substances, there is also final causation, in which mind and body concur toward the accomplishment of human nature (*phusis*). In other words, beyond the “functioning” of nature, there is also a teleological approach tightly related to it that expands our views on causation and also our views on mind and consciousness and their relationship to the body. The 4E cognition, with its embodied/embedded/enactive/extended views on the human mind, goes in the same direction. Moreover, even if we do not manage here to completely escape the “explanatory gap” (Levine, 1983)—for instance, by adopting a non-physicalist metaphysical framework such as the process theory (Prentner, 2018)—we can still offer a more comprehensive view of physicalism, which would support scientific research, instead of denying its cognitive and philosophical value and impact.

All this is clearly related to Aristotle's definition of the soul as the form of the body, which pervades not only his biological treatises, but also his entire work, and is very close to the embodied/embedded/enactive/extended mind postulated by the supporters of the 4E cognition. Moreover, even if this account of the human soul or mind does not offer a complete account of phenomenal consciousness, it is still useful to better understand some forms of cognition and some aspects of phenomenal consciousness, as will be explained in the following sections of the article.

I would still need to clarify from the very outset though that I am not focusing here on an “Aristotle vs. Descartes” account of consciousness. That would not be of much use in the given context. Moreover, their different concepts of soul, mind, substance, etc., and their different methodologies have already been thoroughly explained by numerous and revered scholars throughout the years. I am trying to just propose a 4E cognition cognitive and philosophical context for those neuroscientists studying neurophysiological phenomena underlying consciousness and willing to work together with cognitivists and philosophers of mind in an interdisciplinary manner. Moreover, I am trying to show that this 4E cognition context may be inspired by both ancient philosophical views such as those of Aristotle and contemporary scientific work, which makes it quite a wide and flexible context indeed. By doing so, I am in no way trying to present the 4E cognition as the ultimate cognitive/philosophical context for the study of consciousness or to minimize other outstanding attempts at explaining consciousness as related to experience or at analyzing the pitfalls philosophers and neuroscientists may confront during their common interdisciplinary work (cf. Dennett, 2005; Massimini and Tononi, 2018). I am just trying to add a new piece to the conceptual puzzle developed around the scientific study of consciousness (many thanks to one of the anonymous reviewers for suggesting these clarifications).

## 4E cognition, Aristotle, and neurophysiological phenomena related to (visual) consciousness

The paradigm of the so-called 4E cognition (embodied/embedded/enactive/extended) argues for the causal role of both the body and its natural, social, and cultural environments in human cognition (Clark and Chalmers, 1998; Chemero, 2009; Robbins and Aydede, 2009; Menary, 2010; Shapiro, 2011). By that, it challenges the view of cognition based on mental representations, considered to be just symbolic structures channeling contents (Fodor and Pylyshyn, 1988; Hutchins, 1995). In contrast to the mental representations approach, 4E cognition claims that internal representations cannot be independent of bodily (non-neural) actions and their functional details and that propositional knowledge cannot be independent of motor programmes. In this respect, 4E cognition has developed alongside research programmes in reactive or behavior-based robotics—those programmes that trained artificial intelligence within a dynamic interaction with the world in the 1980s and 1990s and started from the assumption that robots should not be devised only as “walking encyclopedias” as minds were not only used “for thinking” but also “for doing” (Clark, 1997).

There are also various everyday life phenomena that have determined specialists in cognitive science to go in the direction of 4E cognition. For instance, human beings sometimes gesture during conversations and that helps them better communicate—that is, better process both language and information (McNeill, 1992). Moreover, when trying to remember something or to perform other cognitive tasks, human beings may use part of their bodies or their environment in order to simplify information processing (Donald, 1991)—for instance, fingers when counting.

Then, at the turn of the century, there was a new discovery in neuroscience that encouraged the development of 4E cognition even more: the so-called “mirror neurons,” which activate not only when humans perform specific actions but also when they see others perform these actions or simply when they think of these actions (Gallese, 2003; Rizzolatti and Craighero, 2004; Iacoboni, 2011). That has developed into a specific branch of research throughout the years and was related to yet another discovery: on the one hand, vision guides action and, on the other hand, the feedback generated by bodily movement is important for visual processing and visual consciousness (O'Regan and Noë, 2001).

The study of visual consciousness actually became one of the empirical research domains that substantially enhanced 4E cognition and was in turn supported by it (Gibbs, 2006 offers a list of many other applications of 4E cognition, but, for the sake of clarity and due to the limited space ascribed to this article, I will only concentrate on the example of visual consciousness). Formerly, visual consciousness was analyzed as a process within the brain (Crick and Koch, 1990; Crick, 1996; Chalmers, 2000; Metzinger, 2000; Koch, 2004). However, the relationship between conscious visual experience and the neurophysiological phenomena related to it has always been both empirically and philosophically puzzling. Throughout time, the matching-content thesis (the perfect match between a specific conscious experience and the neurophysiological phenomena related to it) and the minimal substrate thesis (the so-called neural correlates of consciousness—NCCs), in both the same person at various times and various persons at the same time, have become more and more challenged by the 4E cognition supporters (O'Regan and Noë, 2001; Thompson and Varela, 2001; Noë, 2004; Noë and Thompson, 2004).

While not considering the 4E cognition proponents' views as instances of absolute truth and while acknowledging that many of their views have already been suggested by previous philosophers and cognitivists, I think it is still worth noting how they argued on the basis of two specific cases in which the subjects of an experiment can overlook even obvious changes in a visual scene: change-blindness (Levin and Simons, 1997) and inattentional blindness (Mack and Rock, 1998). The traditional view of the brain reconstructing detailed internal models of the visual field was seriously challenged by these experiments and the view that our visual consciousness also depends on sensorimotor contingencies was enhanced, a matter on which the 4E cognition proponents concentrated extensively (Hickerson, 2007; Noë, 2010; Shapiro, 2010; O'Regan, 2011; Loughlin, 2014). They joined the trend that ceased to interpret visual consciousness as a brain process that creates mental models and started viewing it more as a skill that is related to the movements of the agent. Visual processing also came to be considered as an activity that is extended and partially controlled by the agent—that means that agents have to “do” something in order to become visually conscious of some specific object (Gibson, 1979).

Moreover, they have to take the external environment into account as well, an idea possibly inspired by Aristotle's *On the Soul* 3.2 (425b12-18), which makes an interesting point on the issue of visual consciousness^2^. To the question: “How are we conscious that we see?” Aristotle responds that sight is perceiving itself—namely that the same sense must perceive both sight and color, the object of sight. Otherwise, if one supposes a special sense to apprehend what one sees, one must suppose another to apprehend this, and so on—the process would continue *ad infinitum*. In other words, according to Aristotle, either when it perceives the external objects or when it perceives itself while perceiving the external objects, the human mind has to consider both itself and the external environment.

Another important issue that Aristotle discusses in *On the Soul* 3.2 (425b22-26) and may be relevant for the 4E cognition proponents' views on (visual) consciousness is that of sense experience (*phantasia*) as a bridge between body and soul, on the one hand, and between perception and thinking, on the other hand. Aristotle asserts that “to perceive” means to form an opinion corresponding exactly to a direct sense experience. In *On the Soul* 3.3 (427b29-429a9) and 3.7 (431a1-20), this is described as a movement produced by a sensation actively operating. Aristotle insists that, while sense experience is distinct from both sensation and intellect, it forms the link between them by prolonging and recording the former and thus making memory and recollection possible by supplying it with illustrations. Moreover, as in *On the Soul* 3.2 (425b22-26), it is also a prerequisite for appetite as each sense organ is receptive to the perceived object even without its matter; that is, even when the objects of perception are gone, sensations (*aisthêseis*) and information from the senses (*phantasiai*) are still present in the sense organ.

Similarly, when referring, in *On the Soul* 3.7 (431a1-20), to the practical intellect and especially to practical wisdom (*phronêsis*), which deliberates on ethical issues, Aristotle explains that the soul never thinks without recollected information from the senses because this takes the place of direct experience for the thinking soul. Then, in *On Memory and Recollection* 1 (449b31-450a1, 450a23-24), he insists once more that it is impossible to think (*noein*) without perceptions (*phantasma*) and that memory itself is related to that. Moreover, in *On Memory and Recollection* 2 (453a15-23), recollection induces bodily reactions as it searches for mental pictures in the physical sphere. Aristotle gives here an example of the annoyance some may show when they cannot remember something. In such a case, he suggests, they set in motion a bodily part in which the “affection” resides. Moreover, these are again views that seem to have inspired some of the 4E cognition conceptions mentioned above.

To offer more details, I would note here that, in tune with Aristotle's views, for the 4E cognition proponents, bodily movements seem to be important in the creation of concepts. Moreover, such an idea is also in tune with those put forward by neuroscientists asserting that thinking about objects implies multimodal activations of previous experiences and neurophysiological phenomena related to perception and action regarding the conceptualized objects. This challenges once more the mere representation of concepts through abstract symbols and the definition of cognition only in terms of mental representations. Indeed, humans seem to construct concepts through different modalities in various contexts (Solomon and Barsalou, 2001; Gallese and Lakoff, 2005; Beauchamp and Martin, 2007). Emotions also seem to be represented by concepts that are embedded in bodily feelings and behavior re-enactivations. Moreover, these could be simple facial expressions or gestures or more complex movements (Niedenthal et al., 2014; Oosterwijk and Barrett, 2014).

The 4E cognition proponents generally conclude that two people with the same neurophysiological phenomena related to visual consciousness, but placed in different environments, would have different conscious experiences. “A brain in a vat” (BIV), without any bodily input, as the homonymous thought experiment postulates it to be (Block, 2005), would not have any visual experience due to the fact that it would not be able to interact with the environment. Sensory processing and the ability to control one's bodily movements, the so-called “sensorimotor coupling with the environment,” are thus mandatory for visual conscious experience (Farrer and Frith, 2002; Farrer et al., 2003; Haggard, 2005; Tsakiris et al., 2007). Moreover, that perfectly fits the neuroscientists' demonstration that consciousness and action seem to sometimes recruit the same neural networks used in the interaction with the environment (Frith et al., 2000; Leube et al., 2003). Sensorimotor circuits are thus used not only for controlling one's body but also for the formation of concepts related to visual consciousness. This overlap challenges the traditional hypothesis of the localization of cognitive functions (Anderson, 2010).

Another network recruitment that challenges the localization of cognitive functions and strengthens the above argument referring to visual consciousness can be found in the relationship between consciousness and pathology—for instance, epilepsy. The dynamics of conscious states with respect to epileptic activity is an interaction between two functional networks: one physiological—the default mode network (DMN)—and one pathological—the epileptome (Donos et al., 2016; Maliia et al., 2016). Two competing theories seem to be at work here: “the network inhibition” hypothesis argues that there is an indirect inhibition of the DMN via the profound diencephalic structures (thalamus) (Blumenfeld et al., 2004; Blumenfeld, 2015), while the “diminished workspace” hypothesis suggests that during the epileptic seizure, more and more critical hubs of the DMN connectome are recruited by the epileptome (Fahoum et al., 2013). However, different from the motor and conceptual network recruitment contributing to the visual consciousness of the healthy brain, the one in the pathological brain seems to happen in a temporal sequence, not at the same time. It is important to note though, in relation to the idea of non-localized cognitive functions in the pathologic brain, that the interictal connectivity analysis used to describe the functional networks behind this complex dynamics demonstrates that the seizure onset zone (SOZ) in epilepsy is not a localized, but a dynamic one, engaging in a variety of network configurations that can be accurately described only in a personalized manner (Donos et al., 2016; Maliia et al., 2016).

For that reason, a Riemannian manifold, in which the brain network dynamics are figured out by clustering methods, and a temporal segmentation process sometimes applied to refine the segments for SOZ localization seem to represent a good method to interpret multiple-channel iEEG signals, in which the ictal process involves continuous changes of information propagation (Qi et al., 2019). A similarly subtle and flexible methodology is applied at the Complex Mind Lab of the University of Jaén (Spain), in which the Departments of Psychology and Experimental Biology are involved. For data modeling and analysis of brain activity, the specialists of the Complex Mind Lab use non-linear analyses of the EEG signal when working with patients displaying various other alterations of consciousness (Ibáñez-Molina and Iglesias-Parro, 2014, Esteban et al., 2018; Ruiz de Miras et al., 2019; Soler-Toscano et al., 2022; Iglesias-Parro et al., 2023).

In short, such important discoveries in neuroscience during the last decades, referring to both healthy and pathological human brain, were paralleled by substantial changes in philosophy and cognitive science. The 4E cognition proponents represent a significant part of this wave of change in the philosophical methodology for the study of the mind, which has thus become more integrated into the broader interdisciplinary field of cognitive science (Kahane et al., 2012; Knobe, 2015).

Within this context, beyond the important part played by sensorimotor activity and environment in cognitive processing, 4E cognition specialists also offered new insights on consciousness as related to prosociality and empathy—especially to the folk psychology debates regarding humans' mindreading capacities, that is, the capacities of predicting and explaining the actions and emotions of other humans (Stüber, 2006, 2012). This is also connected to the distinction between two types of empathy: “basic and reenactive,” on the one hand, and “mirroring and reconstructive,” on the other hand (Goldman, 2006, 2011). While the first refers to the “mirror neurons” (Gallese, 2003; Rizzolatti and Craighero, 2004; Rizzolatti and Sinigaglia, 2008; Iacoboni, 2011), the second refers to a kind of “mindreading,” in which we understand one another's behavior and emotions in complex social contexts, while complex neurophysiological phenomena and neuronal areas such as the medial prefrontal cortex, temporoparietal cortex, and the cingulate cortex get involved (Frith and Frith, 2003; Kain and Perner, 2003).

But this also goes much further and beyond 4E cognition and its possible inspiration in some of Aristotle's views, on the one hand, and in the latest discoveries on (visual) consciousness in neuroscience, on the other hand. For instance, the idea that a better understanding of the body underlines conscious experience and intersubjectivity appeared already in Husserl's phenomenology (Gallagher, 2005, 2009, 2012; Gallagher and Zahavi, 2008). Moreover, it was under the influence of Merleau-Ponty's phenomenological views that cognitivists started emphasizing the importance of the structural brain–body–world coupling against the traditional computationalist views (Varela et al., 1991). This was related to the notion of autopoiesis in biology. Moreover, by describing living systems as active, adaptive, self-maintaining, and self-individuating, the idea of autopoiesis inspired enactivist views in cognitive science in turn (Maturana and Varela, 1998; Di Paolo and Thompson, 2014). The latter themselves are important for the explanation of consciousness and of various human processes and behaviors (Noë, 2004; O'Regan, 2011).

In fact, a few more clarifications are necessary here in relation to the 4E cognition proponents: while the embodied cognition approach developed primarily in relation to biology, that of extended cognition was more philosophically informed—especially via discussions on functionalism and individualism (Clark and Chalmers, 1998). However, despite some differences ensuing from that, they do inform and support each other, as in the case of Clark's view that the active embodiment of cognition is an argument for the extended mind thesis (Clark, 2008). There are indeed critics of this coupling of embodied and extended cognition and they argue that it results from confusion or from ignoring the differences between the “causes” and the “constituents” of cognition (Aizawa, 2007; Adams and Aizawa, 2008; Adams, 2010). Lately, cognitivists managed nevertheless to reach a consensus regarding the association of embodied and extended cognition views. Action-guiding visual processes were again, as above, at the center of explaining 4E cognition as follows: visual processes guide actions via visual information, but only when this is naturally coupled and functionally integrated with bodily activities and these actively embodied visual processes are also extended (Wilson, 2004), a view that is again in tune with Aristotle's views mentioned above in this section of the article.

## 4E cognition and the “mind–body problem”: some more considerations

In fact, this article started from the assumption that cognitive science and the philosophy of mind can and should help overcome such differences and make the research processes fluently ensue from one another and cross-fertilize one another, beyond various differences of focus or methodology. Moreover, it also started from the assumption that the philosophical conceptual analysis should also be supported by a historical analysis in this respect. One can better understand and transcend all differences when noticing the historical fact that many of the problems in our research on brain networks and consciousness nowadays go back to Descartes's dualism—that is, to the concepts of unextended mind (*res cogitans*) and extended matter (*res extensa*). The perpetuation and interpretation of this dualism in both philosophy and science until nowadays sometimes played their part in impeding empirical research—at least at a subliminal level if not also at a rational and methodological one as well.

And, as it is often the case, placing ideas in their proper historical and hermeneutical context may considerably help solve the matter. For instance, looking at the ancient Aristotelian views on the “soul” (its empirical substratum, emotions, consciousness, and agency) may somewhat help us widen our perspective and place the Cartesian model in its proper context. We may thus understand that Descartes' views were just a moment in the history of science and cognition and that it was not only that various modern and contemporary philosophers criticized Descartes for his mind/body dualism, but also numerous other philosophers before Descartes, including important ancient ones, had completely different views on the human brain, mind, and consciousness. Moreover, one should not overlook Descartes' own theory of individuation, which seriously undermines any reductionist interpretation of Descartes' consciousness as the defining characteristic of his unextended mind and totally opposed to extended matter, to which it is purportedly superior and does not benefit much from interacting with it (Wilkes, 1992; Magee, 2003).

In my view, in order to transcend “Descartes' error” (in António Damásio's words—cf. Damásio, 1994) of ignoring the physiology of rational thought or decision and the emotions as conveyors of information and guidance coming from the body to the rational thought, we should have a brief look again at Aristotle's views on the relationships between mind and body in cognition. These views had a lasting aftermath—they are indeed still discussed in comparison to those of Descartes nowadays (Kahn, 2005; Charles, 2008)—but I think a few more details should be added on their possible relationship to the 4E cognition approach. For instance, an important idea to mention here would be that Aristotle's “passions of the soul,” including emotions and desire, were often considered psycho-physical and represented an alternative to Cartesian and post-Cartesian views throughout the recent history of philosophy (cf. Charles, 2008 discussing Aristotle's *On the Soul*).

And even more important, in this context, is to note that Clark and Chalmers (1998), in *The Extended Mind*, consider the separation between mind, body, and environment an unprincipled distinction and propose a kind of “coupled system,” in which the environment should be functioning as a part of the mind. The main criterion mentioned by Clark and Chalmers in support of such an extended cognitive system is thus the “functioning” of the external objects with the same “purpose” as the internal processes. By that, Clark and Chalmers actually use the same main criteria Aristotle himself used when discussing the relationship between mind, body, and nature. The importance of “functioning” and “purpose” in the 4E cognition approach thus justifies once more the focus on Aristotle's own concept of cognition in the present article.

Actually, the connection between “functioning” and “purpose” has already been discussed by several scholars in relation to Aristotle's teleology in the biological works and especially in his *Parts of Animals*. Two types of teleology (from the Gr. *telos*, which can be translated as “purpose”) are considered to be involved here: a primary kind referring to the realization of an internal, pre-existing potential for form and a secondary one referring to the emergence of “functions” as the result of both formal and material causation (Gotthelf, 1976–77, 1987; Leunissen, 2010a,b). Moreover, this type of interpretation is clearly in tune with 4E cognition. Studying a few forms of 4E cognition in relation to a few Aristotelian concepts and their aftermath in the history of philosophy/cognitive science may thus clarify some details in the evolution of the 4E cognition and its interest in the neurophysiological phenomena underlying consciousness.

For instance, the specific concept of mind Aristotle may have actually envisaged is still debated, but it is clear though that it does not fit a rationalist cognitive model based on mental representations. In fact, Aristotle seems to have initiated some of the non-reductive materialist views later developed by Putnam and Nussbaum (1995), Charles (1984), p. 197–250; and Charles (2008), p. 1–2 and fn. 1–2, in which the functional and teleological aspects noted above were emphasized. Moreover, while doing that, Aristotle asked the same question Descartes would ask much later: How are psychological activities or their descriptions related to physical processes? Aristotle's answers to these questions clearly seem to be quite in tune with the contemporary 4E cognition ones—especially when Aristotle discusses psychological properties as possibly supervening on or emerging from physical ones (Charles, 2008, p. 2–3, especially footnotes 3–4 and, for more details, Sorabji, 1974, 2001; Everson, 1997; Caston, 2005, p. 267–268).

While Descartes' views on mind and body have often been quoted as a source for the idea of the would-be “explanatory gap” (Levine, 1983, p. 354–361) regarding consciousness—the fact that conscious subjective experience accompanies specific functions of the brain and the neural/computational mechanisms behind them without being completely explained in terms of these functions/mechanisms—we can try to discuss Aristotle's views as possible precursors of some 4E cognition views. The mind–body problem can be thus studied from completely different angles within an Aristotelian frame of reference and that would help us avoid the property dualism deemed the “hard” problem of consciousness in cognitive science—that is, the autonomy of consciousness from the physical properties upon which it supervenes (Chalmers, 2010). As noted in the introduction, an Aristotelian context for the mind–body problem would also be a subtler and more encompassing paradigm than the Cartesian one. Moreover, here I will concentrate on two conceptions that pervaded Aristotle's work and puzzled interpreters over the years, but can particularly shed some light on the so-called “mind–body problem”: (1) causality in nature (*phusis*), on the one hand, and (2) the active intellect (*nous poiêtikos*), on the other hand.

## Aristotle's causality in nature

Regarding causality in nature (*phusis*), although Aristotle talks everywhere of a nature that (in tune with his teleological approach) acts regularly, methodically, and in a manner that art also does, with the best result as its final goal, he never definitively states whether this nature is corporeal or incorporeal, substantial or accidental (Zekl, 1987). For instance, in his *Physics*, nature is either an inner impulse to movement, or some unshaped material, or form, or even a transcendental principle. Sometimes, nature as the form of a thing is actually the purpose (*telos*) toward which it develops. Other times, nature as matter is the means to the end (Ross, 1923, p. 67–71). For an additional explanation of this rather ambiguous concept of nature, one should look at Aristotle's three different approaches to discussing causes (the semantic approach in *Physics* 2.3, the physical-metaphysical one in *Parts of Animals* 1.1, and the logical-epistemic one in *Posterior Analytics* 1.2) (Duhot, 1989, p. 21–24). The type of knowledge that the human mind can acquire in the context of such an ambiguously defined concept of nature can be thus somewhat explained by Aristotle's views on causality in nature although all this still remains rather difficult to define. One can clearly notice, nevertheless, the hints to a type of cognition that is embodied, embedded, enactive, and extended (the 4E cognition nowadays).

Some clarification may be provided by *Physics* 2.3 (194b16-195b30 and in particular 195a21-27), where Aristotle presents the four causes while suggesting two pairs of oppositions: that between the material and formal causes and that between the efficient and final causes, all related to the same two ideas of “functioning” and “purpose” mentioned above as related to 4E cognition. The first opposition represents a static ontological point of view and the second a dynamic one. Then, in *Parts of Animals* 1.1 (639b11), only two causes are considered for the explanation of nature and of the way the phenomena are produced: the final and the efficient, only the dynamic aspect being here at stake.

Within this context, while movement from within may represent the distinction between natural and manufactured objects in Aristotle, reason may actually represent the link between them. Moreover, that happens because art or manufacture (*technê*), as an imitation of nature (*phusis*), requires knowledge of both form and matter as it studies both the end and the means (Ross, 1923, p. 66–70). The procedure of nature is, in this respect, assimilated to that of art, and the study of nature is included among the constructive or manufacturing sciences rather than the theoretical ones in *Parts of Animals* 1.1 (639b16-21). Moreover, the mind dealing with all these is indeed extended in order to grasp both matter and form and re-produce them in the “craft.” It is thus extended especially because it is related to “purpose” and searching for the “functions” of the parts—the same idea mentioned above and related to 4E cognition.

Furthermore, in *Physics* 2.5 (196b17-25), Aristotle explains that, besides things that exist out of necessity, there are also others defined by finality and the latter may display finality by either thought or nature. This double causality remains ambiguous, nevertheless. For instance, when Aristotle describes the structure of animals as the result of purpose in *On Heavens* 1.7 (271a 33), the question that may appear is: Whose purpose? Nature is generally described as acting for a purpose, but nature is not a conscious agent; it is only the vital force present in all living things. This vital force is indeed connected to the *psuchê*, to the soul as the vital force of the body, but here as elsewhere, Aristotle seems to have been content (as many thinkers later inspired by him have also been) with a notion of a purpose that is not the purpose of any particular mind. His teleology does not necessarily imply intentionality (Kullmann, 1979, p. 2). However, whenever the human mind decides to deal with such teleology (even without intentionality in nature), it does need to proceed intentionally nevertheless and also in an extended manner in order to grasp the “vital force” of nature through the specific functions of the parts and the specific purpose of each of them—again in a 4E cognition type of explanation.

Finally, Aristotle's idea of a “configurating” power of nature discussed in *De partibus animalium* 1.1 is strengthened in his *Physics* 2.5 (199b26-33), where the purpose is considered present in nature even if no specific deliberation (*bouleusis*) is present. This can also be compared with Aristotle's idea of the vegetative soul presented in *On the Soul* 2.4 (415a23-25) as that which deals with nutrition (*trophê*) and generation (*genesis*). However, again, in order to grasp this vegetative function of the soul, the higher deliberative function needs to be extended, to be connected to the body and its causal role in cognition. In natural sciences, Aristotle's purpose is thus rather the perpetuation of the type, the preservation of the species (Ross, 1923, p. 125–126, 135–136), but one should also connect this view with the one Aristotle displays in his ethics, where the purpose is happiness (*eudaimonia*), which has a normative significance and requires awareness and knowledge in pursuit of the good. Moreover, neither awareness nor knowledge can be obtained without filling the gap between the “vital force” of nature and its “configurating” power, or between the material and the formal causes—in an interplay with efficient and final causes (mentioned above) that, again, may have inspired the 4E cognition proponents. However, as noted above, all this complex account of causality would not help explain the mind–body problem by itself, without yet one more of the puzzling notions in Aristotle—the active intellect.

## Aristotle's active intellect

In order to explain how the notion of active intellect (*nous poiêtikos*) fits in our discussion here, a few more details on Aristotle's account of the human soul need clarification in the following paragraphs. *On the Soul*, alongside the *Parva Naturalia* (especially *On Sense and Sensible Objects, On Memory and Recollection, On Sleep and Waking, On Dreams, On Respiration*) and *On Breath* treat various topics on the relationship between body and mind that are also related to the active intellect. In the *Parva naturalia* Aristotle is particularly interested in sense and its impact on consciousness and his views in these short treatises are complementary to those in *On the Soul*, where Aristotle tries to outline a systematic and general theory by applying the actuality/potentiality relationship to study the interaction between body and soul or sense/sensible and mind/intelligible. In fact, Aristotle himself was a biologist and the son of a doctor. He believed in experiment and dissection as a means of collecting evidence and his views on the soul were definitely influenced by his knowledge of physiology.

For instance, in *On the Soul* 1.1, Aristotle maintains that his intention is to account for the essence (*ousia*) of the soul as the principle (*archê*) of animal life (402a7-10), which is essential for the study of nature (*phusis*; 402a4-6), the concept already discussed above as a possible source of inspiration for the 4E cognition proponents. Aristotle is especially interested in understanding to which class of entities the soul belongs, what it is—a particular thing (namely, a substance), or a quality, or quantity, or another of the established categories—and whether it has only potential or fully actual existence (402a22-27). He insists that, generally, none of the emotions (or, as he calls them, “affections of the soul”) can exist apart from the body although thinking could exceptionally do that sometimes. However, he also insists that even thinking, when it is dependent on *phantasia* (phenomena or sense experience that facilitate the perception of things as they appear to us, which can be partially assimilated to our contemporary concept of sense experience associated with visual consciousness) cannot exist apart from the body either.

When discussing the “affections of the soul” (i.e., emotions), Aristotle thus defines them as associated with the body (either by being caused by bodily states or by affecting the body) and he illustrates his view by referring to gentleness, fear, pity, courage, and joy, as well as love and hate (403a3-11-403a16-19). Moreover, cognition in Aristotle seems to be embodied as long as he thinks that there is almost no *dianoia* (thinking) without *phantasia* (sense experience), which proves that even thinking (*dianoia*) is somewhat associated with the body (more on emotions and thinking in Aristotle in this respect in Van der Eijk, 2002; Charles, 2008; Mingucci, 2015).

Moreover, one may see as two different interpretations of reality is actually just a methodological distinction. For instance, Aristotle presents two definitions of anger: according to a natural philosopher (*phusikos*), on the one hand, and according to a logician or philosopher trained in discursive argument (*dialektikos*), on the other hand—the first describes the matter (a surging of the blood and heat around the heart) while the second describes the form (craving for retaliation; 403a26-403b3). There are two types of discourse presented here favoring a methodological distinction that doctors and psychologists nowadays still inherit.

Aristotle's account of the soul as summarized in *On the Soul* 2.4 (415a14-416b32) and 2.12 (424a17-424a34) finally suggests that, just like the different discourses on the same reality, the faculties of the soul cannot be considered as separate “parts,” but only as logically separable aspects of vitality, corresponding to different degrees of existence or to different activities in the same degree (nutritive, appetitive, locomotive, sensitive, and intellective). This suggests that, in the last instance, even if they either fall under the sensitive faculty or are shared by it with the intellective faculty, they all resist strict taxonomies and can rather be understood in terms of what we call 4E cognition nowadays.

Aristotle was indeed swaying between the old Homeric tradition, the Ionian physicists or physiologists (as he called them), and the Platonic philosophy (Cairns, 2014). For that reason, his views on the soul and its cognitive capacities (sensory, affective, and intellectual) sometimes developed in the direction of what we now call 4E cognition, in which the body does determine specific forms of knowledge. Moreover, this is one of the reasons for which *On the Soul* received various contradictory interpretations, some more radical than others, even if many of them agree that Aristotle's theory of the soul is an embodied one (Shields, 1988, p. 103–137; Sorabji, 1991, p. 227; Bos, 2003, p. 8; Shields, 2016, p. xvii–xliii).

This was certainly largely understood within natural science or physics, especially in terms of the distinction between motion (*kinêsis*) and activity *(energeia)* in *On the Soul* 3.7 (431a4-7), where perception is considered an activity rather than a motion (Polansky, 2007, xii, referring to Burnyeat, 1995). Other interpretations, also important here, concentrate on the hylomorphic (matter + form) unity of soul and body and the relationship between the soul as actualization (*entelecheia*) of the natural body (*sôma physikon*)—the body being also interpreted as “instrumental” or “tool-like” or “equipped with tool-like parts” (that is, with organs; *organikon*) beyond being potentiality (*dunamis*) (Bos, 2003, p. 6; cf. King, 2007, p. 322-23).

According to such interpretations, in the case of sensation, senses are mere potentialities that are actualized by sensible objects acting upon them. According to *On the Soul* 2.5 (416b32-418a6) and 2.11 (423b27-424a16), the sensitivity of the subject and the sensibility of the object become identified and this actualization is sensation. However, in Aristotle's view, this requires a medium (air, flesh, etc.), which is activated by the sense object and transmits a stimulus to the sense organ, thereby assimilating it with the sense object. Before stimulation, the sense organ must be in a neutral state as any determinate quality would interfere with its receptivity. Every sense is thus a sort of a mean or balance between contraries.

Furthermore, as in *On the Soul* 3.2 (425b27-31), the activity (*energeia*) of the sensible object and of the sensation is one and the same (as it should also be in any description of 4E cognition), even though their essence is not the same. Additional explanations on this appear in the *Parva naturalia* 1 (436b6-8), where Aristotle explains that sensation is produced in the soul through the medium of the body, and as the exercise of sense perception does not belong exclusively either to the soul or to the body (a potentiality and its actuality reside in the same subject and what we call sensation as actuality is a movement of the soul through the agency of the body), it is clear that anything that affects us (for instance, sleep) is peculiar neither to the soul without a body, nor to the body without soul, which is actually not capable of sensation (an idea that appears also in *On Sleep and Waking* 1 (454a7-12).

Now, while also considering Aristotle's general account of change, one can also note that, in *On the Soul* 3.4 (429a13-18), he explains that, as perception involves the reception of a sensible form by a specific sensory faculty, so thinking involves the reception of an intelligible form by a specific intellectual faculty (cf. Sorabji, 1974; Caston, 1996, 2005, 2006, 2008; Everson, 1997; Johansen, 1998; Charles, 2008; Marmodoro, 2014; Shields, 2016). What should be noted though is that, in both cases, there is change by the acquisition of a form by something capable of receiving it—“the perceptive faculty is in potentiality such as the object of perception already is in actuality” and the same happens in the case of thinking.

This hylomorphic analysis of thinking is obviously an extension of the general model of hylomorphic change used by Aristotle in numerous other contexts, but it is important to emphasize here that Aristotle's account of thinking parallels his analysis of perception. Thought is thus a process that is analogous to sensation as it is receptive of form in the same manner that sense is and there are two factors involved in both cases: a passive, material, and indeterminate one and an active and formative one. The intellect is partly passive and partly active as perception itself is. Moreover, that favors again, in my view, the idea that the 4E cognition proponents may have been inspired by Aristotle's account of the soul and its perceptive/thinking faculties [see also Aristotle, *On the Soul* 3.4 (429a13–18) for comparison], with reason, sense, motion, and emotion clearly interrelated in as much as sense is not mere local motion generated from one body to another, or simple resistance of one body to the motion of another, but a cognitive act—either a recognition or an active perception and awareness of these motions of the body.

However, the active intellect, as in *On the Soul* 3.4 (429a10) and 3.5 (430a25) and also in *Metaphysics* 1072b23ff, is separable from the body, impassive, unmixed, and divine in the manner Descartes' *res cogitans* also is while sense can never exist apart from the body. The relationship between the active and the passive intellect or the higher and active part (*to noêtikon*) and the lower, passive, or sympathetic part (*to aisthêtikon*) of the soul, or between the rational and the sensory functions, may still remain somewhat unclear in this respect. Moreover, in *On the Soul* 3.5, Aristotle's account of the active intellect seems to introduce a slightly different account of the intellect (*nous*) in general, as a faculty or power (*dunamis*) of the soul (*psuchê*). This new account is also somewhat different from Aristotle's insistence in *On the Soul* 2.1 (413a3-5) (cf. Shields, 2016) that the soul as a whole was not separable from the body.

Thus, the active intellect as described particularly in *On the Soul* 3.5 (430a17–18) as “separate, unaffected and unmixed” and then also as “everlasting” (430a23) may actually contradict my idea that the 4E cognition proponents were much inspired by Aristotle's cognitive theories. A few questions should be still raised though in this respect: How could the active intellect be separable if it is a capacity of the soul and the soul is not separable? In what sense is the active intellect separable: conceptually or ontologically? And then, from what is the active intellect separable: the body, the other faculties of the soul, some other unspecified category? And, in fact, what exactly is the active intellect (*nous poiêtikos*) and how is it related to the simply unqualified notion of the intellect (*nous*) discussed in other chapters of Aristotle's *On the Soul*? Such questions have raised an entire commentary tradition from antiquity to the present day. Moreover, when addressing them, Aristotle's interpreters present quite diverging answers.

The most generally accepted interpretation (Ross, 1923; Hicks, 1965; Wedin, 1988; Caston, 1999, p. 199; Shields, 2016) is that, as, according to Aristotle, in all nature, everything is in both potentiality (like matter) and actuality (like a craft, giving a specific form to the matter), such differentiation should be present in his account of the soul as well. To exemplify the difference between intellect as it comes to be all things and intellect as an active craft (*poiêtikon*) imposing a specific form to the matter, Aristotle uses its comparison with light, which makes colors actually exist while without light they would just potentially exist. By that, while again highlighting the isomorphism and tight relationship between sensation and thinking, Aristotle also highlights the active capacities of the perceiver and contrasts them with the merely passive capacities of lifeless matter. Thus, the active and creative (like a craft) intellect is not necessarily so far removed from 4E cognition, as one may be tempted to think at first sight, but this still remains an issue to be further discussed—for reasons of limited space available—in a different article.

## Discussion

As noted above, 4E cognition developed in cognitive science and the philosophy of mind alongside research programmes in reactive or behavior-based robotics and started from the assumption that minds were not only used “for thinking,” but also “for doing” (Clark, 1997). By that, from the very beginning, 4E cognition was in tune with science in general and with the scientific research on the brain in particular. Neuroscientists can benefit from this cognitive and philosophical approach, which offers them a broad and flexible philosophical and cognitive research context that could inspire them in their own general accounts and interpretations of multiple empirical data they operate with.

Beyond the technical information and the algorithms they use or develop for better interpreting the data they obtain in their research, neuroscientists also need such interdisciplinary hypotheses or explanations of the human mind and cognition in order to make sense of their findings at the highest and most general level possible. As I have seen at the Complex Mind Lab of the University of Jaén, neuroscientists work intensively in order to find algorithms that can provide a more subtle and enriching explanation and interpretation of their data. They realize that, as an EEG signal, for instance, needs proper, subtle, and multimodal frames of reference and new methodologies for interpretation, so do many of their questions and explanations of the empirical data, which are sometimes in need of philosophical and cognitive suggestions on the so-called “mind–body problem” and on many other similar issues. That happens especially when neuroscientists study human consciousness and the complex brain network dynamics possibly enabling and supporting it. Interdisciplinarity is thus an important requirement for a valuable account of the possible interplay between the neurophysiological phenomena related to consciousness or conscious agency—especially when complex brain network dynamics are involved.

This interdisciplinarity can develop in various directions. Biology, physics, and chemistry are mandatory fields of research when discussing the human mind and consciousness alongside their neurophysiological substratum. However, cognitive science and philosophy cannot be ignored either. Moreover, while being rather blocked by the Cartesian dualism, such research is highly favored by the new 4E cognition paradigm. Besides being in tune with science from its inception, it also offers a wide and flexible approach that is in tune with former valuable accounts of the human mind that influenced both scholarship and scientific research for centuries. Aristotle's account of human cognitive and perceptive faculties in his *On the Soul* is one of these.

In fact, even when taking into account the short discussion at the end of this article on the active intellect, one could hardly think that Aristotle's theory of cognition could favor any abstract rationalism based on mental representations, which would block scientific research on the physiology underlying it. On the contrary, Aristotle was in favor of creating an analogy and connection between sense experience and perception on the one hand and thought on the other hand. In that and many other directions, his explanations resemble those offered by the contemporary 4E cognition, which, in my view, were much inspired by his work or by various later commentaries on it.

For instance, when describing both causality in nature and the actualization of potentiality in the human soul, Aristotle was clearly in favor of an extended type of cognition and a continuous relationship between mind and body. Even when insisting, in *On the Soul* 3.5, on the idea of an active intellect, defined as imperishable, unchangeable, and separable from the body, Aristotle was not so much interested in denying extension, but rather in asserting a creative, craft-like part of the intellect, which actively moulds cognition, by contrast to the passive part of the intellect, which only receives the intellectual forms imprinted on it. Moreover, in the end, Aristotle made it clear that even for the active intellect sensations and perceptions played a direct constitutional, if not always causal, part.

Despite all these, not many scholars were interested in the inspiration the proponents of 4E cognition may have found in Aristotle's or in his commentators' accounts of the human mind. A few significant exceptions have already been pointed out in the notes and the bibliography of this conceptual and historical analysis. But more research needs to be done and this short article is just an attempt to stimulate new interdisciplinary research in this respect. More attention needs to be paid to Aristotle's commentary tradition and to many other aspects of his physiological works as well. They were influential over the years and, even if they may not be relevant from a scientific point of view anymore, they are still relevant for the way we collect and interpret scientific data and even for our own possible biases caused by philosophy or cognitive approaches (such as the Cartesian dualism) in collecting and interpreting such data.

By studying conceptual and historical issues related to the way consciousness and its relationship with its neurophysiological substratum were interpreted over time, we may gain new insights into the way our minds and own views were formatted by university education (where we once studied such matters), by our own readings and interests or by the views of colleagues from other fields of study. This, alongside clear empirical data and scientific theories, may help us become aware of biases that may slow down our research interpretations and may open new paths of interpretation and new ways of calibrating our own research and interpretation algorithms. Moreover, while such interdisciplinary research may have its own limitations because we still need to work out a common language and a common conceptual basis on which to operate, the 4E cognition, which has already developed alongside the latest scientific discoveries on cognition and its neurophysiological substratum and also seems to have been inspired by ancient philosophical traditions, may be a perfect candidate for a philosophical and cognitive context in which to study consciousness in a more comprehensive manner. It is also broad and flexible enough to be able to account for the complexity of human consciousness and of the brain networks supporting it to a quite large extent.

## Author contributions

The author confirms being the sole contributor of this work and has approved it for publication.

## Footnotes

^1^(Many more details on Aristotle's concept of the soul regarding the intelligible/sensible distinction can be found in a large number of works, from ancient Aristotle commentators such as Themistius (317-388 A.D.) (Themistius, translation 1996) to contemporary ones like: Jaeger, 1923; Nuyens, 1939; Barnes, 1972; Gill, 1991, 1997; Granger, 1996 etc.)

^2^(All citations from Aristotle's works are according to the Bekker numbering, the standard form of citing Aristotle, initiated by the Prussian Academy of Sciences complete works of Aristotle, the *Aristotelis opera* (1830-1870), edited by Bekker et al. The English translations of various works of Aristotle are listed in the bibliography just for comparison. They also abide by the Bekker standard form of citation.)
